# Enhanced thermotolerance via 22-nt small RNA-mediated silencing of SMXL4 and SMXL5

**DOI:** 10.1093/plcell/koae239

**Published:** 2024-08-21

**Authors:** Peng Liu

**Affiliations:** Assistant Features Editor, The Plant Cell, American Society of Plant Biologists; Donald Danforth Plant Science Center, Saint Louis, MO 63132, USA

Small RNAs are crucial regulators involved in various biological processes. Among them, 21-, 22-, and 24-nt small interfering RNAs (siRNAs) can all trigger gene silencing (Sigman et al. 2021). However, the study of endogenous 22-nt siRNAs has been challenging due to their limited abundance. Researchers using Arabidopsis mutants defective in both cytoplasmic RNA decay and 21-nt siRNA production were able to observe an accumulation of 22-nt siRNAs (Wu et al. 2020). In a new study, **Yajie Pan and colleagues (Pan et al. 2024)** investigated the *super-killer2* (*ski2*) *dicer-like4* (*dcl4*) double mutant and found that 22-nt siRNAs enhance plant thermotolerance mainly by repressing *SUPPRESSOR OF MAX2 1-LIKE4* (*SMXL4*) and *SMXL5* genes.

Ski2 mediates cytoplasmic RNA decay, while DCL4 is responsible for producing 21-nt siRNAs. The loss of these functions in the *ski2 dcl4* double mutant led to an accumulation of 22-nt siRNAs from endogenous genes on both strands and elevated expression levels of numerous genes. Since siRNA usually inhibits gene expression, these genes may be induced as a secondary effect caused by siRNA activity. Gene ontology (GO) analysis indicated that most of these upregulated genes are involved in stress responses, including various heat-responsive genes (HRGs). While wild-type seedlings die when grown at 44 °C, about 70% of *ski2 dcl4* seedlings survive, indicating enhanced thermotolerance. The production of 22-nt siRNAs depends on DCL2. In the *ski2 dcl4 dcl2* triple mutant, both the enhanced thermotolerance and 22-nt siRNA accumulation observed in the *ski2 dcl4* double mutant are restored to wild-type levels, suggesting that DCL2-dependent 22-nt siRNAs contribute to this enhanced thermotolerance.

Previous studies have shown that many genes generate 22-nt siRNAs in the *ski2 dcl4* double mutant, with SMXL4 and SMXL5 among those generating the highest quantities of 22-nt siRNAs (Wu et al. 2020). In the new work, Pan and colleagues found that the *smxl4 smxl5* double mutant exhibits remarkable thermotolerance, with altered expression levels of numerous HRGs similar to those in the *ski2 dcl4* mutant (Pan et al. 2024). In the *ski2 dcl4* mutant, *SMXL4* and *SMXL5* expression is downregulated but restored to wild-type levels in the *ski2 dcl4 dcl2* triple mutant, indicating that their inhibition depends on 22-nt siRNA. Additionally, heat stress reduces *SMXL4* and *SMXL5* transcript levels in the wild type. These findings suggest that SMXL4 and SMXL5 act as negative regulators of plant thermotolerance.

*HEAT SHOCK TRANSCRIPTION FACTOR A2* (*HSFA2*) is a master regulator of plant thermotolerance by transcriptionally regulating HRGs (Charng et al. 2007). Pan and colleagues found *HSFA2* expression to be upregulated in the *ski2 dcl4* and *smxl4 smxl5* mutant compared with the wild type. The authors further showed that overexpression of SMXL4 or SMXL5 alone represses transcription from the *HSFA2* promoter, and both proteins bind directly to the *HSFA2* promoter. A *smxl4 smxl5 hsfa2* triple mutant abolished the thermotolerance phenotype of the *smxl4 smxl5* double mutant, suggesting that HSFA2 acts downstream of SMXL4 and SMXL5 in the heat stress response pathway.

*HSFA1d* and *HSFA1e* are transcription factors that directly regulate *HSFA2* expression (Nishizawa et al. 2006). Pan et al. showed that SMXL4 and SMXL5 block this activity under normal conditions (Figure, A; Pan et al. 2024). SMXL4 interacts with HSFA1d and HSFA1e, as well as with SMXL5. Through this direct or indirect association with the *HSFA2* promoter region, SMXL4 and SMXL5 repress the transcription of *HSFA2*. Under heat stress conditions, reduced expression of SMXL4/SMXL5 relieves HSFA1d/A1e repression, leading to increased *HSFA2* transcription (Figure, B).

**Figure.**
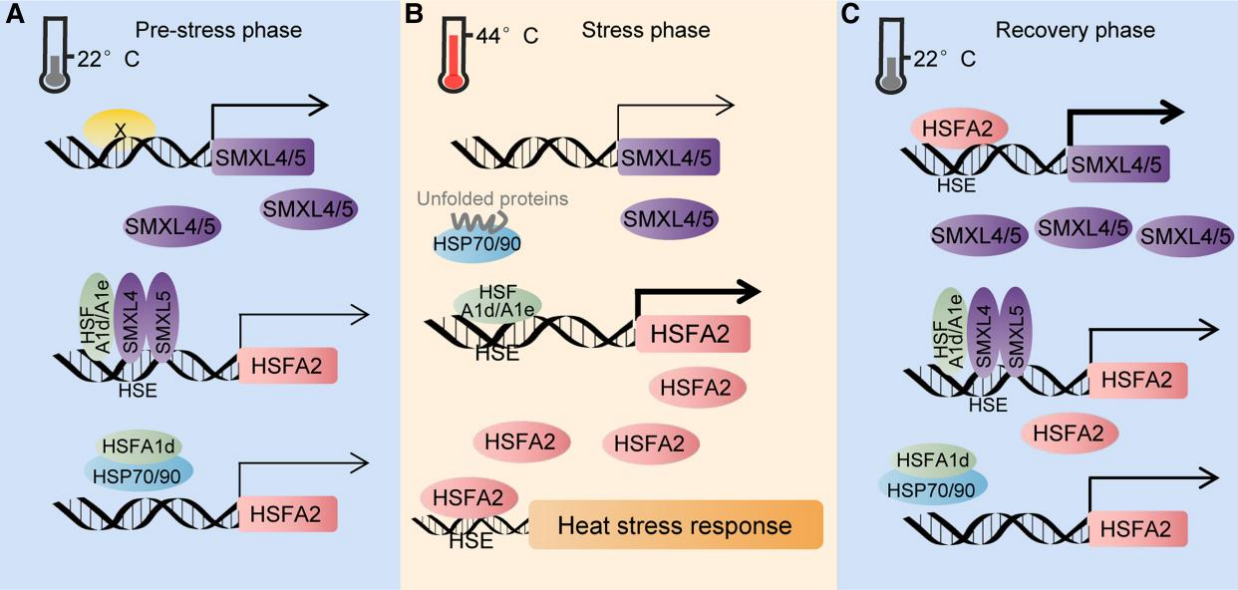
Model for SMXL4/SMXL5-mediated inhibition of thermotolerance. **A)** Under normal conditions, SMXL4/SMXL5 bind to the *HSFA2* promoter and interact with HSFA1d/A1e to inhibit their activity. **B)** In high temperatures, reduced expression of SMXL4/SMXL5 (mediated by 22-nt siRNAs) relieves HSFA1d/A1e repression, leading to increased *HSFA2* transcription and activation of the heat stress response. **C)** During recovery from heat acclimation, HSFA2 increases SMXL4/SMXL5 expression, which then suppresses HSFA1d/A1e, shutting down *HSFA2* transcription and aiding recovery from heat stress. Reprinted from Pan et al. (2024), Figure 8.

Acclimation is a process by which plants adapt to environmental changes under nonlethal stress conditions, enhancing their tolerance to future stressors. In Arabidopsis, heat acclimation stimulates the expression of HSFA2, which in turn activates the transcription of SMXL4 and SMXL5 by recognizing a heat shock element. Subsequently, SMXL4 and SMXL5 inhibit HSFA2 transcription, forming a regulatory feedback loop after thermal acclimation (Figure, C). The study conducted by Pan et al. thus provides new insight into the mechanisms of thermotolerance in plants, highlighting a complex regulatory network involving siRNAs, transcription factors, and protein–protein interactions.

## Dive Curated Terms

The following phenotypic, genotypic, and functional terms are of significance to the work described in this paper:

HSFA2 Gramene: AT2G26150
HSFA2 Araport: AT2G26150

dcl2 Gramene: AT3G03300
dcl2 Araport: AT3G03300

dcl4 Gramene: AT5G20320
dcl4 Araport: AT5G20320

SMXL4 Gramene: AT4G29920
SMXL4 Araport: AT4G29920

SMXL5 Gramene: AT5G57130
SMXL5 Araport: AT5G57130
